# Want a Law for Aseptic Midwifery

**Published:** 1904-10

**Authors:** 


WANT A LAW FOR ASEPTIC MIDWIFERY.—The Potter County Medical Society and the Plains Medical Association (meeting at Amarillo) endorsed a bill submitted by Dr. T. F. McGee to enforce asepsis and antisepsis in all midwifery cases, and appointed Drs. McGee and Lockett, of Amarillo, and Dr. Albert, of Childress, a committee to have same printed and copies sent to every medical society in Texas, and urge them to request their respective representatives in the Legislature to vote for the same. We are glad to see such interest manifested in the cause of preventive medicine, but think the energy in this case is misdirected. The State Association has a Committee on Public Policy and Legislation, who are entrusted with the work of securing a State Board of Health, and each county society has a similar committee auxiliary to the State Association committee. This important matter should be entrusted to these committees. A law for a State Board should comprehend all such details as the above; that is, such a board could enforce the practice of asepsis without a special act of the Legislature.

